# In-House 3D Planning for Corrective Osteotomy of the Distal Radius: Prospective Cohort Study With 1-Year Radiological and Clinical Follow-Up

**DOI:** 10.2196/101764

**Published:** 2026-09-09

**Authors:** Charlotte Stor Swinkels, Peter Axelsson, Katleen Libberecht, Per Fredrikson, Emilia Gryska, Anders Björkman

**Affiliations:** 1 Department of Hand Surgery, Institute of Clinical Sciences, Sahlgrenska Academy, University of Gothenburg, Medicinaregatan 3, Göteborg, Västra Götaland, 413 90, Sweden, 46 31-786 00 00; 2 Department of Medical Physics and Biomedical Engineering, Sahlgrenska University Hospital, Gothenburg, Sweden; 3 Department of Hand Surgery, Sahlgrenska University Hospital, Mölndal, Sweden

**Keywords:** 3D, virtual surgical planning, surgical guides, patient specific, distal radius, in-house

## Abstract

**Background:**

Distal radius malunions occur in 5% to 17% of fractures and may lead to pain and functional impairment. While 3D surgical planning with patient-specific guides has demonstrated high accuracy and reduced fluoroscopy use compared with conventional 2D techniques, its routine clinical implementation remains limited, particularly for in-house hospital-based workflows.

**Objective:**

This study aimed to evaluate the precision of corrective osteotomy for distal radius malunions using in-house–designed 3D patient-specific surgical guides 1 year after surgery. Secondary objectives were to assess clinical and patient-reported outcomes and document intraoperative fluoroscopy use.

**Methods:**

Corrective osteotomies of extra-articular distal radius malunions were performed in 16 consecutive patients using in-house 3D surgical planning and patient-specific surgical guides. Accuracy was evaluated by comparing preoperative plans with 1-year postoperative computed tomography–based 3D models, assessing ulnar variance, volar tilt, and radial inclination. Acceptable error margins were defined as 5° or less for volar tilt and 2 mm or less for ulnar variance. Clinical outcomes were evaluated using patient-reported outcome measures and range of motion, lift, grip, and torque strength measurements.

**Results:**

The mean absolute error was 1 (SD 0.8) mm in ulnar variance, 4.9° (SD 2.7°) in volar tilt, and 1.9° (SD 3.6°) in radial inclination. The corrections were within predefined equivalence bounds of the virtual plans in terms of ulnar variance but not volar tilt. Significant improvements were observed in Patient-Rated Wrist Evaluation (PRWE) and Disabilities of the Arm, Shoulder, and Hand (DASH) scores; pain during activity; pain at rest; lift strength; wrist flexion; and ulnar deviation. In total, 12.5% (2/16) of the patients experienced complications following concomitant ulnar shortening procedures and were transferred to standard care. The median intraoperative fluoroscopy duration was 56 (IQR 26‐72) seconds.

**Conclusions:**

In-house 3D planning was feasible and associated with generally favorable radiological and clinical outcomes at 1 year. However, the predefined equivalence criterion was not achieved for volar tilt, and the findings should be interpreted considering the small sample size and observed complications. In-house 3D planning supports continuous surgical refinement and quality improvement.

## Introduction

Distal radius fractures are the most common type of fractures in the upper extremities. In 2024, wrist fractures made up 17% of all fractures registered in the Swedish Fracture Register [1]. While most distal radius fractures heal with satisfactory alignment [2], approximately 5% to 17% result in malunion, which may cause functional impairment and pain [3-5]. Previous studies have demonstrated that accurate anatomical correction of distal radius malunions leads to better clinical outcomes [5-8].

Traditional corrective techniques using 2D radiographic planning and freehand osteotomy are limited in their ability to achieve precise restoration of the anatomy [5,9]. To improve surgical precision, 3D surgical planning and patient-specific surgical guides are increasingly used in complex skeletal reconstructions. Studies applying this approach to distal radius malunions report high precision with small residual errors [10-17] and reduced use of intraoperative fluoroscopy [6]. Although 3D planning has been in clinical use for over 15 years and evidence supporting its effectiveness continues to grow, its routine integration into clinical practice is currently limited.

Two main models exist for the clinical implementation of 3D virtual planning and patient-specific surgical guides. The standard approach involves outsourcing the planning and production of guides to commercial providers. However, this model requires transferring patient data to external partners and restricts direct collaboration between surgeons and engineers, with high costs being an additional limitation. An alternative is to establish an in-house 3D planning unit within the hospital. This model facilitates close, continuous collaboration between surgeons and engineers, potentially improving planning accuracy and cost efficiency. However, it also requires dedicated resources, staff training, and a robust quality management system to ensure clinical reliability and safety. In our previous preclinical study [18], the same patient cases underwent independent in-house and commercially provided 3D planning. The resulting surgical guides were evaluated on 3D-printed bone models, and the in-house workflow demonstrated noninferiority to the commercial workflow. The present study extends these findings by evaluating the surgical accuracy and 1-year clinical and radiological outcomes after clinical implementation of the same in-house planning workflow.

The primary aim of this study was to assess the precision of corrective osteotomy of malunited distal radius fractures 1 year after surgery using in-house–designed 3D patient-specific surgical guides. Secondary aims were to assess clinical and patient-reported outcome measures (PROMs) to determine whether using patient-specific surgical guides leads to improvements in function and/or satisfaction 1 year after surgery compared to before surgery. An additional aim was to document the use of intraoperative fluoroscopy.

## Methods

### Study Design and Population

A total of 16 consecutive adult patients requiring corrective osteotomy for a symptomatic extra-articular distal radius malunion were prospectively enrolled over a 2-year period from July 2021 to July 2023. The median age of the case series (n=14, 87.5% women and n=2, 12.5% men) was 61 (range 22‐77) years. Exclusion criteria were bilateral distal radius deformity, cognitive impairment, psychiatric illness, active substance abuse, inability to read and understand the language, and any condition known to impair bone healing. The indication for surgery was pain in most patients (n=11, 68.8%). A total of 18.8% (n=3) of the patients experienced loss of range of motion (ROM), but only one of them had this as the only indication for surgery. In addition to corrective osteotomy of the distal radius, 18.8% (n=3) of the patients underwent concomitant ulnar shortening to avoid excessive distraction of the distal radius. The ulnar shortening procedures were also performed using 3D surgical planning and guides. A total of 6.3% (n=1) of the patients withdrew before surgery, and an additional 12.5% (n=2) of the patients were excluded from the final analysis after developing nonunion following a concurrent ulnar shortening procedure. These patients were transferred to standard clinical care and underwent revision fixation of the ulna using longer plates. Because they did not complete the study-specific follow-up, they were excluded from the final analysis.

### Ethical Considerations

This study was approved by the Swedish Ethical Review Authority (2021-01974) and performed in accordance with the Declaration of Helsinki. All patients received written and oral information about the study and provided written informed consent prior to enrollment (all participants were adults; therefore, a legal guardian was not required).

### 3D Surgical Planning and Design of Patient-Specific Surgical Guides

**Figure 1.**
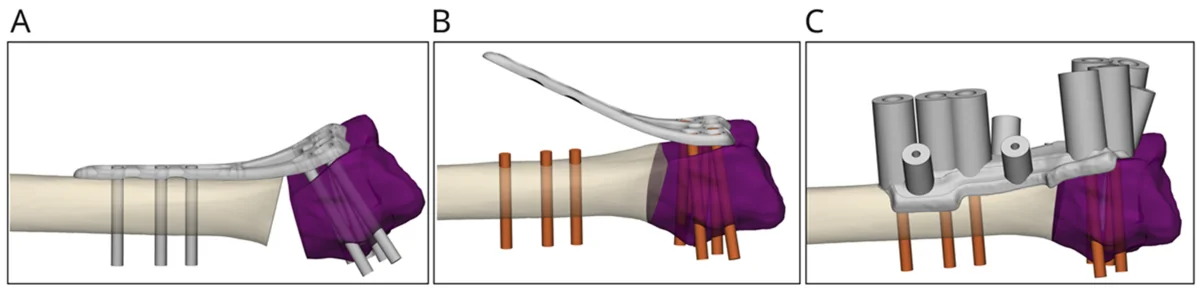
Construction of patient-specific surgical guides: virtual surgical planning (A); the distal bone fragment and osteotomy plate, including distal screws, reversed to the preoperative position (B); and guide design based on planned screw positioning (orange; C).

Each patient underwent bilateral high-resolution computed tomography of the entire forearm using a standardized scanning protocol (100 kVp, 0.625-mm slice thickness, and convolution kernel: bone). Bilateral 3D virtual models of the forearm bones were created by segmenting the patients’ Digital Imaging and Communications in Medicine images using the Mimics Medical software (Materialise NV). The uninjured forearm was mirrored, aligned at the proximal part of the radius, and used as a template. The maximum planned distraction was limited to approximately 12 to 14 mm. This threshold was used as a technical planning constraint based on clinical experience and published recommendations suggesting that concomitant ulnar shortening should be considered in patients with radial shortening exceeding approximately 12 mm [19]. It should not be interpreted as a formally validated cutoff. The amount of ulnar shortening was then planned to achieve bone contact in the radius osteotomy after fixation. Surgical planning and guide design were conducted in-house by a hand surgeon (KL) and a clinical engineer (CSS) using the 3-matic Medical software (Materialise NV) according to previously described methods [18,20] (Figure 1). Digital files of the in-house–designed guides were sent to a subcontractor (Prototal UK) to be 3D printed in accordance with the medical device manufacturing standard ISO 13485. The printing technology used was selective laser sintering on a 3D printer (EOS P 396; EOS GmbH) with a biocompatible polyamide raw material (PA 2200; EOS GmbH). The guides were quality controlled by the hospital’s clinical engineers upon delivery from the subcontractor, repacked, labeled, and sterilized using autoclave according to the hospital’s clinical routines (134°; 4 minutes). The complete manufacturing process was conducted in accordance with the European Union Medical Device Regulation 2017/745.

### Surgical Procedure

**Figure 2.**
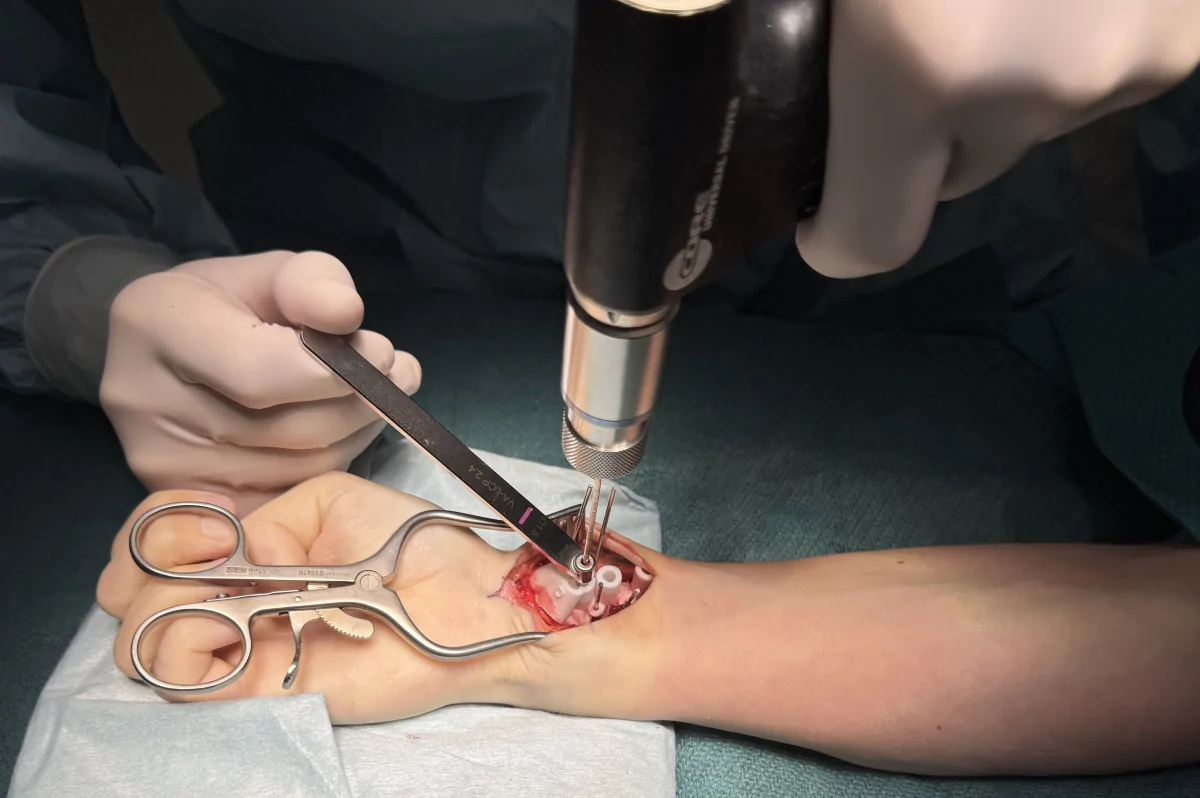
Intraoperative photograph of predrilling screw holes using the surgical guide’s cylindrical sleeves.

All surgeries were performed by 2 senior hand surgery consultants (PA and KL) with experience level 4 according to Tang and Giddins [21]. Both surgeons had prior experience using externally purchased surgical guides. Patients were operated on under general anesthesia, regional anesthesia (brachial plexus block), or a combination of both. After exposure of the volar surface of the radius, the first drilling guide was placed on the bone and fixed with four 1.5-mm K-wires. Proper placement of the first guide was verified using a flat elevator to confirm continuous contact between the guide and the bone surface. The screw holes were predrilled using the guide’s cylindrical sleeves (Figure 2).

When all holes were drilled, the 2 parallel K-wires were left in the bone, and the other 2 K-wires were removed to allow for removal of the drill guide by sliding it off the remaining K-wires. The cutting guide was then positioned by sliding it down to the bone over the 2 parallel K-wires, and the osteotomy was carried out using a power saw. To fixate the bone, either a Variable Angle LCP Two-Column Plate (DePuy Synthes) or a VariAx 2 Distal Radius Plate (Stryker) was used depending on best anatomical fit during the 3D planning phase. The plate was fixated to the distal fragment first with locking screws and then reduced to the radius shaft using the previously drilled holes. If the osteotomy gap lacked bone contact, it was filled with iliac crest graft or residual bone from a concomitant ulnar shortening. If volar bone contact was present, no bone graft was used. Following wound closure, the wrist was immobilized for 2 weeks in a plaster cast. The duration of fluoroscopy use (seconds) was recorded by the radiographic fluoroscopy unit in the surgical room.

### Rehabilitation

Two weeks postoperatively, the cast was replaced with a removable wrist brace, and patients initiated gentle wrist ROM exercises 4 times daily. Light loading was permitted beginning at 4 weeks. After 6 weeks, brace use was typically discontinued, and full wrist loading was allowed provided that radiographic evidence confirmed initial bone healing.

### Primary Outcome: Radiological Measurements

**Figure 3.**
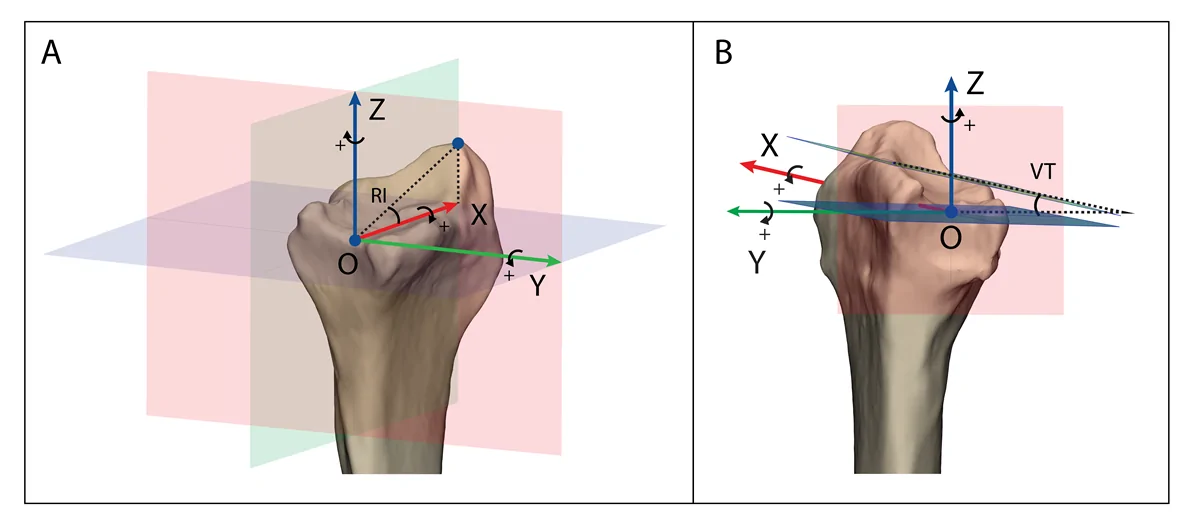
Anatomical reference system and measurement of clinical parameters. “RI” refers to the angle between the x-y plane and the line from O to the radial styloid (A). “VT” refers to volar tilt, the angle between the x-y plane and the plane through the dorsal and volar rim tips (B). The system was adapted for left and right anatomy.

The primary outcome we report is the discrepancy between the 1-year postoperative computed tomography scan and the 3D virtual plan. Volar tilt, ulnar variance, and radial inclination were selected as clinically relevant outcome parameters. On the basis of prior literature, acceptable error margins were predefined as 5° or less for volar tilt and 2 mm or less for ulnar variance [3-5,22]. No specific error margins have been defined for radial inclination as previous studies did not find a significant correlation between radiographic measurements and clinical outcomes [23,24]. Using the 3-matic Medical software, an anatomical reference system was established to ensure clinical interpretability (Figures 3A and 3B) and described in our previous study [18]. Briefly, the origin (0, 0, 0) was positioned at the center of the rim between the lunate fossa and the sigmoid notch, similar to the central reference point used in standard radiographic assessments [18]. The central axis of the radius was defined as the intersection of 2 planes created using a datum-plane function (“two points, perpendicular to view”). The 2 points were manually placed on the radial shaft, one at 30 mm and one at 50 mm proximal to the central reference point using the anteroposterior and lateral views, respectively. The z-axis was aligned parallel to the radial shaft axis at the origin, the x-axis was directed toward the projected tip of the radial styloid and perpendicular to the z-axis, and the y-axis was defined as perpendicular to the x-z plane. Within this reference framework, rotation around the x-axis represented volar tilt, rotation around the y-axis corresponded to radial inclination, and translation along the z-axis represented ulnar variance.

The postoperative and planned radius models were registered in 2 stages. First, the proximal radius was used as the stable reference. Corresponding regions covering approximately the proximal half of the radius were manually selected on the planned and postoperative models using a brush selection tool. The postoperative model was then registered to the planned model using an iterative closest-point algorithm restricted to the selected proximal surfaces.

Following this proximal registration, the coordinate system was reset to the anatomical reference frame of the virtual plan. Corresponding articular regions of the distal fragment were then manually selected on both models, and a second iterative closest-point registration was performed. The transformation between the planned and postoperative positions of the distal fragment was exported as a homogeneous transformation matrix from which the translational and rotational errors were calculated. A transformation matrix shows translation and rotation differences between the planned and postoperative 3D models in all 3 spatial dimensions (x, y, and z).

### Secondary Outcomes: Patient-Reported and Clinical Outcome Measurements

PROM data were collected using the Disabilities of the Arm, Shoulder, and Hand (DASH) questionnaire [25]; the Patient-Rated Wrist Evaluation (PRWE); and the 100-mm visual analog scale for pain at rest and pain during activity. The total resulting scores from both the DASH and PRWE measures range from 0 to 100, where 0 indicates no disability or pain and 100 indicates the worst possible pain or disability [26].

Clinical outcome measurements were assessed preoperatively and 1 year postoperatively. Wrist ROM in extension, flexion, supination, pronation, radial deviation, and ulnar deviation was assessed using a goniometer. Grip strength was measured using a dynamometer (Jamar STD) [27]. Torque (ie, strength during forearm rotation) was measured in kilograms and converted into newton-meters using a dynamometer with a handle attached to a wall using a connection plate [28]. Lifting strength with one arm was measured in kilograms in a neutral wrist position using a hanging scale dynamometer (HCB 50K20; Kern & Sohn GmbH) [28]. Before strength testing, patients were allowed to familiarize themselves with the dynamometer and the testing procedure. One measurement of each ROM and strength outcome was recorded at each assessment. The same measurement equipment was used throughout the study. Measurements were not repeated or averaged, and intraobserver reliability was therefore not assessed. The measurements were performed by a highly experienced hand surgeon (PA; level 4) [21] following the Swedish national manual for measurements in hand surgery [27].

### Data Analysis

The equivalence bounds were defined as mean volar tilt error within –5° to +5° and mean ulnar variance error within –2 mm to +2 mm, representing the clinically acceptable margins based on previous literature. To assess whether postoperative radiological outcomes were equivalent to the preoperative virtual plans (ideal=0° or 0 mm), an equivalence test was performed using the two one-sided tests (TOST) procedure using JASP (University of Amsterdam). Equivalence testing uses TOST at an α value of .05 to assess whether the effect is neither too low nor too high. Combining these 2 tests yields a 90% CI, representing values that satisfy both 1-sided tests of 5%. Equivalence could be claimed if both TOST were significant, meaning that the entire 90% CI of the mean postoperative deviation lay within the defined intervals for volar tilt (−5°, +5°) and ulnar variance (−2 mm, +2 mm). Equivalence testing was performed using the signed differences between the planned and achieved outcomes to assess whether the mean directional error was contained within the predefined lower and upper equivalence bounds. In addition, mean absolute errors were calculated and reported descriptively to quantify the magnitude of the deviations irrespective of their direction (positive or negative).

Due to the small sample size, the secondary outcomes were reported using medians and IQRs, and a nonparametric Wilcoxon signed-rank test was applied to assess the differences between preoperative and postoperative measurements. A *P* value below .05 was considered statistically significant. No corrections for multiple comparisons were applied to secondary outcomes as they were considered exploratory.

## Results

### Radiological Patient Outcomes

**Figure 4.**
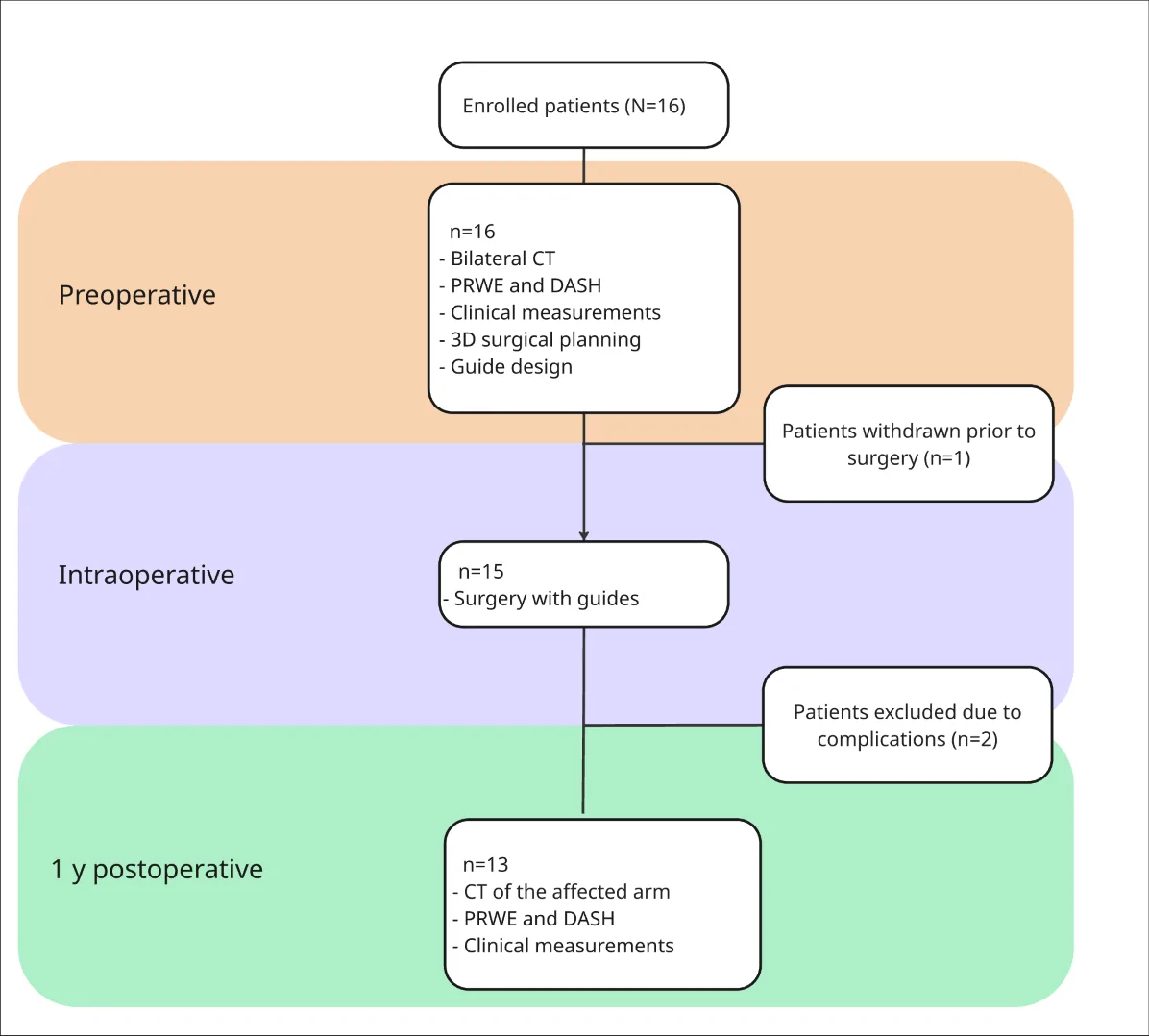
Study flowchart of patient inclusion and data collection. CT: computed tomography; DASH: Disabilities of the Arm, Shoulder, and Hand; PRWE: Patient-Rated Wrist Evaluation.

**Figure 5.**
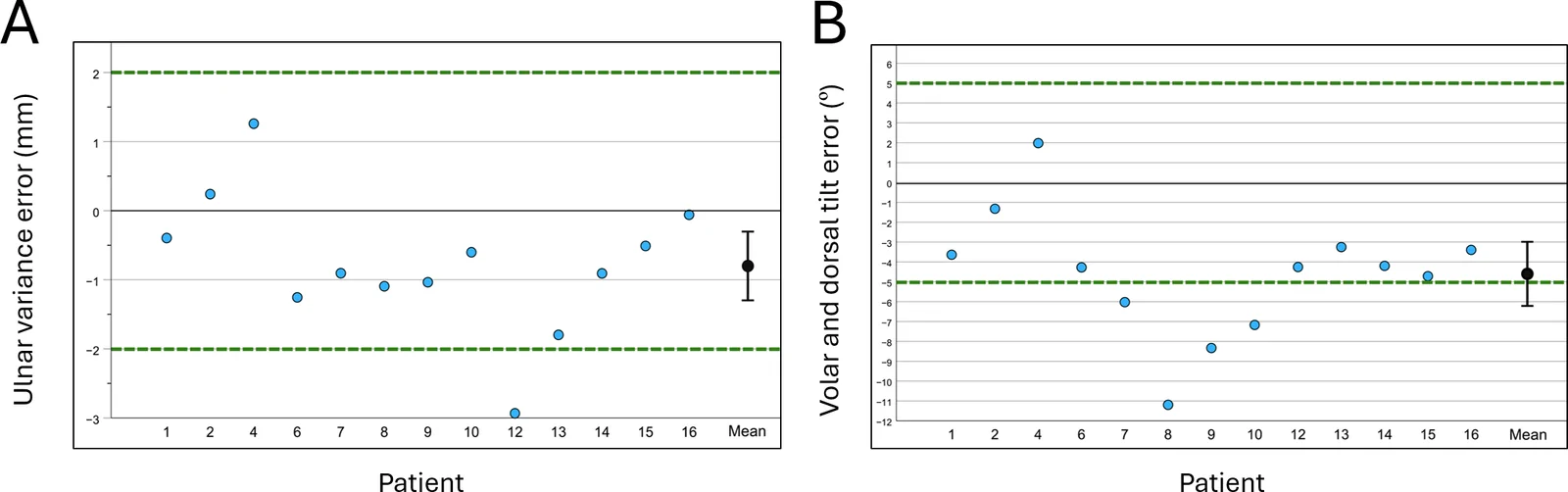
Signed ulnar variance error (A) and volar and dorsal tilt error (B) per patient case and mean (with 90% CI) based on the equivalence test. Clinically relevant and equivalence error margins are marked with a green dashed line (n=13).

For the radiological analysis, of the initial 16 patients, 13 (81.3%) were included (n=1, 6.3% withdrew prior to surgery, and n=2, 12.5% were moved to standard clinical care due to complications). A supplementary table summarizing the available sample size for each outcome and the reasons for missing data is provided in Multimedia Appendix 1, and a study flowchart is shown in Figure 4. An equivalence test was calculated based on the signed values and showed that the error in ulnar variance was considered equivalent to the preoperative plan. Both the lower (–2 mm) and upper (+2 mm) equivalence bounds were statistically significant (*P*<.001), and the 90% CI for the mean error was included in the equivalence boundaries. The signed mean error in volar tilt was not considered equivalent to the preoperative plan. The test against the upper bound (+5°) was significant (*P*<.001), but the test against the lower equivalence bound (−5°) was not (*P*=.33). Thus, the 90% CI for the mean deviation extended beyond the equivalence boundaries. Patient-level errors in ulnar variance and volar tilt, together with the cohort-level mean errors and their 90% CIs used for the equivalence analyses, are shown in Figures 5A and 5B.

**Figure 6.**
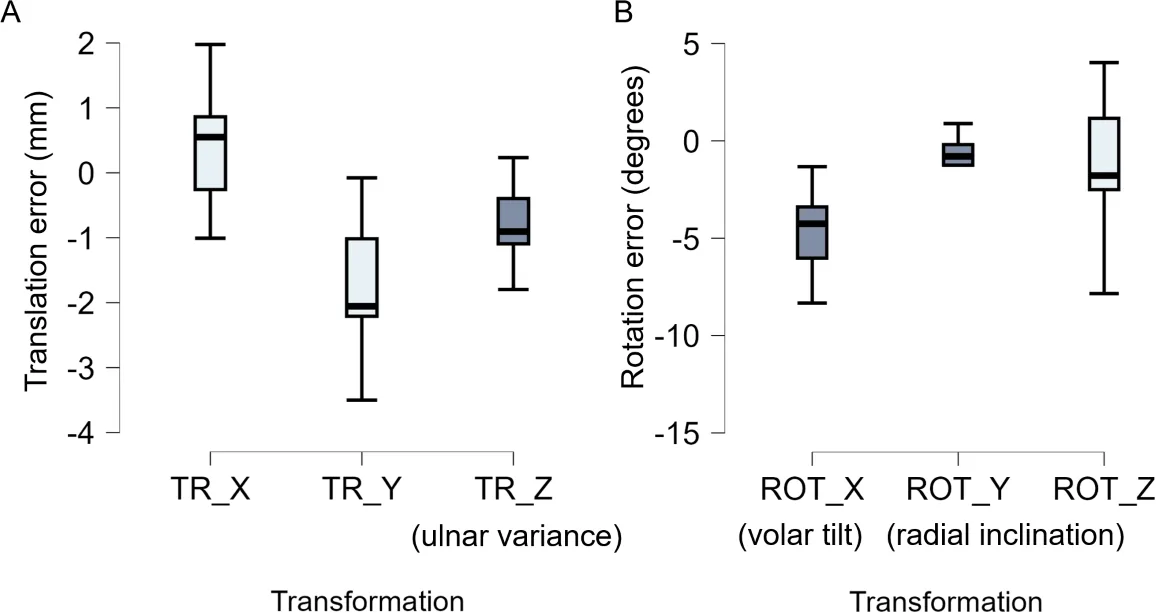
Box plots of the signed mean error between the virtual plan and the postoperative models: translation error (TR) in the x, y, and z directions (A) and rotation error (ROT) in the x, y, and z directions (B; n=13).

Mean absolute errors, representing the magnitude of deviation irrespective of direction, were analyzed for all translational and rotational components. The mean absolute translational errors were below 2 mm in all directions. Among the rotational components, the largest mean absolute error was observed for rotation corresponding to volar tilt. Detailed results for all 6 components are shown in Table 1. Box plots of the signed mean errors in all transformation directions are shown in Figures 6A and 6B.

**Table 1.** Mean absolute errors in all directions between the virtual plan and the postoperative models.

Absolute translational and rotational errors	Values, mean (SD)
Translation X-direction (mm)	0.8 (0.5)
Translation Y-direction (mm)	1.8 (1.0)
Ulnar variance (mm)	1.0 (0.8)
Volar tilt (degrees)	4.9 (2.7)
Radial inclination (degrees)	1.9 (1.7)
Rotation Z-direction (degrees)	4.0 (3.6)

### PROMs

Preoperative and postoperative PROMs are shown in Table 2. PRWE and DASH scores were analyzed for the same patients included in the radiological analysis (n=13). Pain at rest and pain during activity were analyzed in 76.9% (10/13) of the patients because preoperative data were missing for an additional 23.1% (3/13). Numerical improvements from baseline were observed in PRWE, pain at rest, and pain at activity in all patients except 1 (9/10, 90%). DASH scores improved in all patients except 2 (8/10, 80%). These counts represent any reduction in score and should not necessarily be interpreted as clinically meaningful improvement. Statistically significant improvements were noted in all measurements (*P*<.05 in all cases).

**Table 2.** Medians and IQRs of the pre- and postoperative patient-reported outcome measurements.

	Patients, n	Preoperative, median (IQR)	Postoperative, median (IQR)	*P* value
PRWE^a^ (0-100)	13	70 (60‐77)	38 (11-57)^b^	.003
DASH^c^ (0-100)	13	49 (27‐58)	23 (8-36)^b^	.03
Pain at rest (0-100)	10	51 (15‐76)	14 (1-20)^b^	.02
Pain at activity (0-100)	10	72 (53‐82)	36 (11-47)^b^	.007

a PRWE: Patient-Rated Wrist Evaluation.

b The difference was statistically significant (*P*<.05).

c DASH: Disabilities of the Arm, Shoulder, and Hand.

### Clinical Outcome Measurements

Preoperative and postoperative clinical outcome measurements are shown in Table 3. Lifting and grip strength were analyzed for the same patients included in the radiological analysis (n=13). Torque measurements were analyzed in 92.3% (12/13) of these patients because preoperative values were missing for 7.7% (1/13). A statistically significant improvement in lifting strength was observed postoperatively compared to preoperatively in 66.7% (8/12) of the patients. The remaining strength measurements were not statistically significant.

The ROM data for the affected arm both pre- and postoperatively are shown in Table 4.

**Table 3.** Medians and IQRs of the pre- and postoperative clinical outcome measurements.

	Patients, n	Preoperative, median (IQR)	Postoperative, median (IQR)	*P* value
Lifting strength in a neutral position (kg)	13	6.2 (4.2‐9.1)	9.9 (6.2-12.3)^a^	.04
Grip strength (kg)	13	14.0 (6.0‐23.0)	22.0 (12.5‐30.0)	.15
Torque supination (Nm)	12	2.9 (1.9‐4.4)	3.8 (2.9‐4.5)	.13
Torque pronation (Nm)	12	2.0 (1.2‐2.4)	2.2 (1.4‐2.8)	.78

a The difference was statistically significant (*P*<.05).

**Table 4.** Range of motion of the affected arm pre- and postoperatively (n=13).

	Preoperative, median (IQR)	Postoperative, median (IQR)	*P* value
Extension (degrees)	60 (45‐70)	60 (53‐68)	.44
Flexion (degrees)	45 (30‐60)	60 (50-63)^a^	.003
Supination (degrees)	75 (60‐90)	70 (65‐88)	.61
Pronation (degrees)	65 (60‐70)	70 (63‐80)	.33
Radial deviation (degrees)	17 (15‐25)	20 (15‐30)	.22
Ulnar deviation (degrees)	30 (25‐35)	40 (40-48)^a^	.008

a The difference was statistically significant (*P*<.05).

Significant improvements in flexion and ulnar deviation were observed postoperatively relative to preoperatively. All patients except 1 (11/12, 91.7%) showed numerical improvement in these measurements. No significant improvement was observed in extension, supination, pronation, and radial deviation.

### Intraoperative Fluoroscopy

The median intraoperative fluoroscopy duration was 56 (IQR 26‐72) seconds (mean 54.9, SD 9.3 seconds) to allow for comparison with other studies. Fluoroscopy data were available for 12 procedures. Two patients who underwent a concomitant ulnar shortening procedure were excluded from the fluoroscopy analysis to avoid including fluoroscopy attributable to the additional procedure. The final fluoroscopy analysis therefore included 10 patients.

### Complications

In our cohort including 15 patients (1/16, 6.3% withdrew prior to surgery), 4 (26.7%) required additional or revision surgery. Two of these patients developed nonunion following the concomitant ulnar shortening procedure and underwent revision fixation of the ulna using longer plates. Because they were transferred to standard clinical care and did not complete the study-specific follow-up, they were excluded from the final analysis of the primary radiological outcome. Nonunion occurred in the ulna for 2 out of 3 patients treated with concomitant ulnar shortening. In one of these patients, screw loosening in the distal radius also occurred, leading to the partial recurrence of radial deformity.

Implant removal and median nerve decompression were performed in one patient, with relief of symptoms. Another patient had persistent ulnar-sided wrist pain and mild symptoms of carpal tunnel syndrome. Resection of the ulnar styloid, arthroscopic shaving of a central triangular fibrocartilage complex tear, and median nerve decompression yielded good results. Additionally, one patient had transient increased chronic pain and returned to the same pain level as prior to surgery.

## Discussion

This prospective study demonstrates that corrective osteotomy of distal radius malunions using in-house 3D surgical planning and patient-specific surgical guides can be performed with generally small deviations between the planned and postoperative correction. However, because the study did not include a control group, no conclusions can be drawn regarding whether this approach is more accurate than conventional 2D planning or other surgical techniques. The results showed equivalence in ulnar variance, whereas differences remained in volar tilt. The mean absolute alignment errors 1 year after surgery compared with the preoperative virtual plans were very low for ulnar variance (1, SD 0.8 mm) and radial inclination (1.9°, SD 3.6°), whereas a higher mean error was observed for volar tilt (4.9°, SD 2.7°), indicating less consistent correction. The additional translational and rotational error components were also evaluated because deviations outside the conventional radiographic planes may have clinical consequences. Translation in the radioulnar and dorsovolar directions may influence mediolateral and anteroposterior positioning of the distal fragment, with potential effects on cortical alignment, distal radioulnar joint congruity, tendon clearance, and implant positioning. Rotation around the longitudinal axis of the radius may alter the rotational alignment of the distal fragment and potentially affect joint congruity and forearm mechanics. In the present study, the mean absolute translational errors were below 2 mm in all directions. Among the rotational components, the largest mean absolute error was observed for the component corresponding to volar tilt. However, clinically meaningful thresholds have not been established for these individual 3D error components, including radial inclination, and their clinical significance therefore cannot be determined definitively from the present data. Further studies should relate these errors to functional outcomes, patient-reported outcomes, and clinically relevant complications.

Upon closer case-level analysis, 38.5% (5/13) of the patients had errors outside the predefined margins. The ulnar variance error exceeded the predefined margin in 7.7% (1/13) of the patients, whereas the volar tilt error exceeded the margin in 30.8% (4/13) of the patients included in the analysis. A possible explanation for the volar tilt errors is the challenge of achieving the planned offset angle between the plate and the bone while using variable-angle screws in patients with reduced bone mineral density. As covered in earlier publications [11,20], an offset between the bone and the plate may be required to achieve anatomical correction using standard volar plates due to suboptimal fit on the malunited distal radius.

All cases except one had less volar tilt than planned. Contributing factors to the negative volar tilt error include soft tissue tension and settling of the radius on the distal locking screws. Volar plates achieve fixation primarily through the volar cortical bone [29] as screws provide limited purchase in the cancellous or dorsal cortex. Consequently, restoration and maintenance of volar tilt depend largely on a single point of fixation in the rotational plane. Our assumption is supported by the results of our prior surgical simulations on plastic bone models, which showed no errors outside the predefined margins in terms of volar tilt and radial inclination [18]. These discrepancies highlight the complexity of surgical procedures. Plastic models cannot replicate the compression forces, soft tissue involvement, and accessibility in the wound.

When comparing our results to those reported in the literature, the mean alignment errors are in line with those of similar studies. In a randomized controlled trial, Buijze et al [6] reported statistically significant improvement of volar tilt and radial inclination correction with 3D vs 2D planning. The residual errors after 3D planning were similar to ours, with residual deformity relative to the contralateral side (volar tilt: 4.1°; radial inclination: 2.2°; ulnar variance: 1.5 mm) [6]. However, their measurements were performed using conventional 2D radiographs. Compared to conventional 2D planning, the mean alignment errors using 3D planning were notably smaller. A study using 3D measurements of 2D planned osteotomies by Vroemen et al [30] found higher errors (volar tilt: −6.2°; radial inclination: 0.3°; ulnar variance: −2.2 mm). The 2D planning group in the study by Buijze et al [6] reported significantly higher errors in volar tilt and radial inclination but similar ulnar variance (volar tilt: 7.4°; radial inclination: 4.9°; ulnar variance: 1.2 mm) [6]. A study using conventional 2D planning by von Campe et al [22] reported that only 6 out of 15 patients satisfied the criteria of a correction within 5° angular deformity and 2-mm ulnar variance compared to the uninjured wrist.

The mean fluoroscopy duration in the present study was 54.9 (SD 9.3) seconds, which was similar to the 57.8 (SD 37.7) seconds reported for the 3D planned group by Buijze et al [6] and lower than the 140.3 (SD 101.4) seconds reported for their conventional 2D planned group. These findings suggest that the in-house 3D planning workflow did not increase fluoroscopy requirements and may reduce fluoroscopy use compared with conventional planning. This indicates that ionizing radiation exposure is reduced for both patients and medical personnel when 3D virtual planning and patient-specific surgical guides are used during corrective osteotomies—a finding that has also been demonstrated in several previous studies [6,31,32].

We observed postoperative improvements in PROM scores, lifting strength, flexion, and ulnar deviation, which were consistent with results reported in other studies [6,8,14]. The median improvements in PRWE and DASH scores were both above the minimum clinically important difference of 11.5 and 14 points, respectively [33]. The significantly improved PROM scores, despite the modest improvement in ROM, could be explained by a significant decrease in pain at rest and pain during activity, which allowed the patients to perform their everyday activities with less pain.

A total of 12.5% (2/16) of the patients required additional surgery, and 12.5% (2/16) required revision surgery due to complications related to ulnar shortening, which is an additional procedure carrying its own risk of complications. After in-depth incident analysis, it was concluded that the ulnar shortening failures were a result of suboptimal fixation in combination with pronounced shortening (5 and 10 mm). Although the complications were not directly related to 3D planning, we decided to return to conventional techniques for performing concomitant ulnar shortening while continuing to use 3D planning for distal radius malunions.

In this study, the cost of in-house planning and 3D-printed surgical guides was approximately €1100 per case (including costs for clinical engineers, surgeons, software licenses, and manufacturing; €1=US $1.17 as of August 27, 2026). The comparable cost from an external commercial provider was €2700 per case. However, the reported costs reflect only the planning and manufacturing workflow and do not include broader treatment-related costs, such as those associated with additional imaging, readmission, implant removal, treatment of nonunion, or revision surgery. These findings should therefore not be interpreted as a comprehensive analysis of total treatment costs.

This study has several limitations. It was not designed as a randomized trial, and it did not include a control group treated with conventional 2D methods. However, comparisons could be made with other studies using 2D and 3D planning methods. Furthermore, the small sample size and missing outcome data reduced the precision and generalizability of the secondary findings and precluded meaningful subgroup analyses, such as comparisons between younger and older patients. The varying number of observations across outcomes may also have introduced attrition bias. Although all procedures were corrections of extra-articular distal radius fracture malunions, there was some heterogeneity as ulnar shortening was performed in 18.8% (3/16) of cases.

Future studies could assess outcomes following technical adjustments, including strategies to compensate for predictable deviations such as loss of volar tilt. By analyzing prior residual errors relative to the planned outcomes of larger datasets, we could better estimate the target volar tilt needed to achieve the desired correction. In addition, as osteoporotic bone tolerates less distraction than nonosteoporotic bone, future research should focus on identifying acceptable distraction limits according to bone quality.

In conclusion, the use of 3D virtual planning and patient-specific surgical guides in corrective osteotomies enables precise evaluation of outcomes by comparing preoperative plans with postoperative results. Residual alignment errors and complications in some cases underscore that, even with advanced planning tools, surgical outcomes remain influenced by patient-specific anatomical and biomechanical factors. Surgeons should be aware of potential insufficient corrections with this technique, particularly in volar tilt, and consider integrating anticipated adjustments during the planning phase. The results underscore the value of an in-house 3D planning team, which facilitates continuous quality improvement and refinement of surgical technique.

## Supplementary material

10.2196/101764 Multimedia Appendix 1 Available sample size for each analysis and reasons for missing data.

## Abbreviations

DASH: Disabilities of the Arm, Shoulder, and Hand
PROM: patient-reported outcome measure
PRWE: Patient-Rated Wrist Evaluation
ROM: range of motion
TOST: two one-sided tests
