# Supplementary material for: In-House 3D Planning for Corrective Osteotomy of the Distal Radius: Prospective Cohort Study With 1-Year Radiological and Clinical Follow-Up
Source: JMIR Perioper Med. 2026 Sep 9;9:e101764. doi: 10.2196/101764 (PMC13557323; doi:10.2196/101764)
Supplement: Supplementary file 1 [file periop-v9-e101764-s001.docx]

**Supplementary information**

Available sample size for each analysis and reason for missing data

| **Analysis** | **Sample size (n)** | **Reason for missing data** |
| --- | --- | --- |
| Initially included study patients | 16 | N/A |
| Radiological outcome, PRWE, DASH, lifting strength, grip strength, Range of Motion | 13 | 1 patient withdrew, 2 patients excluded |
| Pain at rest, pain at activity | 10 | 1 patient withdrew, 2 patients excluded, 3 patients did not fill in the form |
| Torque strength (supination and pronation) | 12 | 1 patient withdrew, 2 patients excluded, 1 patient was not measured preoperatively |
| Fluoroscopy use (sec) | 10 | 1 patient withdrew, data not noted during surgery in 3 patients, data from 2 patients with ulnar shortening procedure was excluded |
